# Aqua­chloridobis[5-(2-pyrid­yl)-1*H*-tetra­zolato-κ*N* ^1^]iron(III)

**DOI:** 10.1107/S160053680902443X

**Published:** 2009-07-01

**Authors:** Bo Wang

**Affiliations:** a Ordered Matter Science Research Center, College of Chemistry and Chemical Engineering, Southeast University, Nanjing 210096, People’s Republic of China

## Abstract

The title compound, [Fe(C_6_H_4_N_5_)_2_Cl(H_2_O)], was synthesized by hydro­thermal reaction of FeCl_3_ with 2-(1*H*-tetra­zol-5-yl)pyridine. The iron(III) metal centre exhibits a distorted octa­hedral coordination geometry provided by four N atoms from two bidentate organic ligands, one water O atom and one chloride anion. The pyridine and tetra­zole rings are nearly coplanar [dihedral angles = 4.32 (15) and 5.04 (14)°]. In the crystal structure, inter­molecular O—H⋯N hydrogen bonds link the complex mol­ecules into a two-dimensional network parallel to (100).

## Related literature

For physical properties such as permittivity, fluorescence, magnetism and optical properties of metal-organic coordination compounds, see: Fu *et al.* (2007 ▶); Huang *et al.* (1999 ▶); Liu *et al.* (1999 ▶); Xie *et al.* (2003 ▶); Zhang *et al.* (2000 ▶, 2001 ▶). For the structure of a related tetra­zole compound, see: Fu *et al.* (2008 ▶).
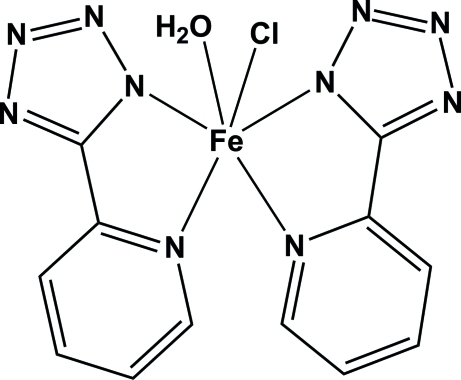


## Experimental

### 

#### Crystal data

[Fe(C_6_H_4_N_5_)_2_Cl(H_2_O)]
*M* *_r_* = 401.60
Monoclinic, 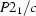

*a* = 17.072 (3) Å
*b* = 7.1905 (14) Å
*c* = 14.292 (3) Å
β = 113.85 (3)°
*V* = 1604.6 (7) Å^3^
*Z* = 4
Mo *K*α radiation
μ = 1.13 mm^−1^
*T* = 298 K
0.15 × 0.10 × 0.10 mm

#### Data collection

Rigaku Mercury2 diffractometer
Absorption correction: multi-scan (*CrystalClear*; Rigaku, 2005 ▶) *T*_min_ = 0.867, *T*_max_ = 0.894
15693 measured reflections
3678 independent reflections
3226 reflections with *I* > 2σ(*I*)
*R*_int_ = 0.040

#### Refinement

*R*[*F* ^2^ > 2σ(*F* ^2^)] = 0.034
*wR*(*F* ^2^) = 0.080
*S* = 1.13
3678 reflections
226 parameters
H-atom parameters constrained
Δρ_max_ = 0.33 e Å^−3^
Δρ_min_ = −0.37 e Å^−3^

### 

Data collection: *CrystalClear* (Rigaku, 2005 ▶); cell refinement: *CrystalClear*; data reduction: *CrystalClear*; program(s) used to solve structure: *SHELXS97* (Sheldrick, 2008 ▶); program(s) used to refine structure: *SHELXL97* (Sheldrick, 2008 ▶); molecular graphics: *SHELXTL/PC* (Sheldrick, 2008 ▶); software used to prepare material for publication: *SHELXTL/PC*.

## Supplementary Material

Crystal structure: contains datablocks I, global. DOI: 10.1107/S160053680902443X/rz2339sup1.cif

Structure factors: contains datablocks I. DOI: 10.1107/S160053680902443X/rz2339Isup2.hkl

Additional supplementary materials:  crystallographic information; 3D view; checkCIF report

## Figures and Tables

**Table 1:** Hydrogen-bond geometry (Å, °)

*D*—H⋯*A*	*D*—H	H⋯*A*	*D*⋯*A*	*D*—H⋯*A*
O1*W*—H1*WA*⋯N4^i^	0.98	1.67	2.652 (2)	178
O1*W*—H1*WB*⋯N9^ii^	0.82	1.80	2.626 (2)	176

Symmetry codes: (i) 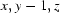; (ii) 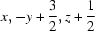.

**Fig. 1.**
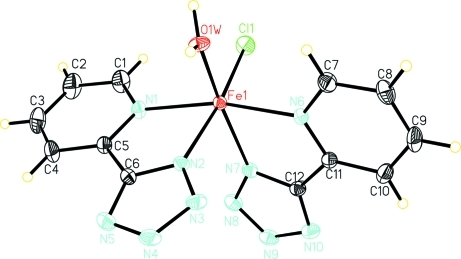
The molecular view of the title compound with the atomic numbering scheme. Displacement ellipsoids are drawn at the 30% probability level.

**Fig. 2.**
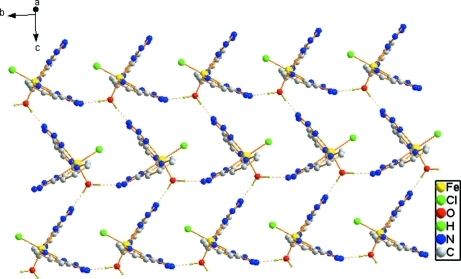
The crystal packing of the title compound viewed along the a axis, showing the two dimensionnal hydrogen bondings network (dashed line). Hydrogen atoms not involved in hydrogen bonding have been omitted for clarity.

## supplementary crystallographic information

### Comment

The construction of metal-organic coordination compounds has attracted much attention owing to their potential propertiess, such as permittivity, fluorescence, magnetism and optical properties. (Fu *et al.*, 2007; Huang *et al.*, 1999; Liu *et al.*, 1999; Xie *et al.*, 2003; Zhang *et al.*,2001; Zhang *et al.*,2000). Tetrazole compounds are a class of excellent ligands for the construction of novel metal-organic frameworks, because of their various coordination modes. (Fu *et al.*, 2008). Herein the crystal structure of the title compound is reported.

In the title compound (Fig. 1), the distorted octahedral coordination geometry around the iron(III) metal centre is provided by four N atoms from two bidentate 2-(1H-tetrazol-5-yl)pyridine ligands, one water O atom and one chloride ion. The pyridine and tetrazole rings are nearly coplanar and only twisted by a dihedral angle of 4.32 (15) and 5.04 (14)°. The geometric parameters of the tetrazole rings are comparable to those observed in a related molecule (Fu *et al.*, 2008). The water molecules are involved in intermolecular O—H···N hydrogen bonds (Table 1) generating a two-dimensional network (Fig. 2).

### Experimental

A mixture of 2-(1H-tetrazol-5-yl)pyridine (0.2 mmol), FeCl_3_ (0.1 mmol), distilled water (1 ml) and a few drops of ethanol sealed in a glass tube was heated at 85 °C. Colourless block crystals suitable for X-ray analysis were obtained after 10 days.

### Refinement

All H atoms attached to C atoms were fixed geometrically and treated as riding with C-H = 0.93 Å with *U*_iso_(H) = 1.2*U*_eq_(C). Water H atoms were located in a difference Fourier map refined as riding, with *U*_iso_(H) = 1.5*U*_eq_(O).

### Crystal data

**Table:**

[Fe(C_6_H_4_N_5_)_2_Cl(H_2_O)]	*F*(000) = 812
*M**_r_* = 401.60	*D*_x_ = 1.662 Mg m^−^^3^
Monoclinic, *P*2_1_/*c*	Mo *K*α radiation, λ = 0.71073 Å
Hall symbol: -P 2ybc	Cell parameters from 3226 reflections
*a* = 17.072 (3) Å	θ = 3.1–27.5°
*b* = 7.1905 (14) Å	µ = 1.13 mm^−^^1^
*c* = 14.292 (3) Å	*T* = 298 K
β = 113.85 (3)°	Block, colourless
*V* = 1604.6 (7) Å^3^	0.15 × 0.10 × 0.10 mm
*Z* = 4	

### Data collection

**Table:**

Rigaku Mercury2 diffractometer	3678 independent reflections
Radiation source: fine-focus sealed tube	3226 reflections with *I* > 2σ(*I*)
graphite	*R*_int_ = 0.040
Detector resolution: 13.6612 pixels mm^-1^	θ_max_ = 27.5°, θ_min_ = 3.1°
CCD profile fitting scans	*h* = −22→22
Absorption correction: multi-scan (*CrystalClear*; Rigaku, 2005)	*k* = −9→9
*T*_min_ = 0.867, *T*_max_ = 0.894	*l* = −18→18
15693 measured reflections	

### Refinement

**Table:**

Refinement on *F*^2^	Primary atom site location: structure-invariant direct methods
Least-squares matrix: full	Secondary atom site location: difference Fourier map
*R*[*F*^2^ > 2σ(*F*^2^)] = 0.034	Hydrogen site location: inferred from neighbouring sites
*wR*(*F*^2^) = 0.080	H-atom parameters constrained
*S* = 1.13	*w* = 1/[σ^2^(*F*_o_^2^) + (0.0265*P*)^2^ + 0.8976*P*] where *P* = (*F*_o_^2^ + 2*F*_c_^2^)/3
3678 reflections	(Δ/σ)_max_ < 0.001
226 parameters	Δρ_max_ = 0.33 e Å^−^^3^
0 restraints	Δρ_min_ = −0.37 e Å^−^^3^

### Special details

**Table:**

Geometry. All esds (except the esd in the dihedral angle between two l.s. planes) are estimated using the full covariance matrix. The cell esds are taken into account individually in the estimation of esds in distances, angles and torsion angles; correlations between esds in cell parameters are only used when they are defined by crystal symmetry. An approximate (isotropic) treatment of cell esds is used for estimating esds involving l.s. planes.
Refinement. Refinement of F^2^ against ALL reflections. The weighted R-factor wR and goodness of fit S are based on F^2^, conventional R-factors R are based on F, with F set to zero for negative F^2^. The threshold expression of F^2^ > 2sigma(F^2^) is used only for calculating R-factors(gt) etc. and is not relevant to the choice of reflections for refinement. R-factors based on F^2^ are statistically about twice as large as those based on F, and R- factors based on ALL data will be even larger.

### Fractional atomic coordinates and isotropic or equivalent isotropic displacement parameters (Å^2^)

**Table:**

	*x*	*y*	*z*	*U*_iso_*/*U*_eq_	
Fe1	0.244652 (16)	0.52062 (4)	0.23914 (2)	0.02111 (9)	
Cl1	0.23959 (4)	0.25457 (8)	0.15658 (4)	0.04015 (15)	
O1W	0.24124 (9)	0.4003 (2)	0.36157 (10)	0.0298 (3)	
H1WA	0.2559	0.2685	0.3733	0.045*	
H1WB	0.2346	0.4617	0.4065	0.045*	
N6	0.10878 (10)	0.5759 (2)	0.17945 (13)	0.0252 (4)	
N2	0.26988 (10)	0.7731 (2)	0.32361 (13)	0.0233 (3)	
N1	0.38334 (10)	0.5417 (2)	0.30588 (13)	0.0277 (4)	
N4	0.28121 (13)	1.0424 (2)	0.39005 (15)	0.0369 (5)	
C11	0.07709 (12)	0.6877 (3)	0.09684 (15)	0.0250 (4)	
N10	0.13543 (12)	0.8769 (3)	−0.00850 (13)	0.0332 (4)	
N3	0.22568 (11)	0.9153 (2)	0.33720 (14)	0.0310 (4)	
N7	0.22328 (10)	0.6973 (2)	0.11379 (13)	0.0260 (4)	
N9	0.21594 (12)	0.8912 (3)	−0.00236 (14)	0.0338 (4)	
N8	0.26901 (11)	0.7850 (3)	0.07022 (14)	0.0324 (4)	
N5	0.36207 (12)	0.9877 (3)	0.41089 (16)	0.0391 (5)	
C5	0.41688 (12)	0.6927 (3)	0.36392 (16)	0.0277 (4)	
C6	0.35228 (13)	0.8211 (3)	0.36874 (15)	0.0266 (4)	
C12	0.14278 (13)	0.7567 (3)	0.06464 (15)	0.0251 (4)	
C10	−0.00933 (13)	0.7286 (3)	0.04887 (17)	0.0345 (5)	
H10A	−0.0299	0.8074	−0.0075	0.041*	
C4	0.50448 (14)	0.7199 (4)	0.41376 (19)	0.0412 (6)	
H4A	0.5264	0.8261	0.4527	0.049*	
C8	−0.03227 (14)	0.5360 (4)	0.1706 (2)	0.0419 (6)	
H8A	−0.0686	0.4823	0.1970	0.050*	
C9	−0.06443 (14)	0.6492 (4)	0.08684 (19)	0.0403 (6)	
H9A	−0.1229	0.6729	0.0555	0.048*	
C7	0.05448 (14)	0.5021 (3)	0.21553 (18)	0.0370 (5)	
H7A	0.0761	0.4255	0.2728	0.044*	
C3	0.55837 (15)	0.5858 (4)	0.4041 (2)	0.0501 (7)	
H3A	0.6174	0.6006	0.4367	0.060*	
C2	0.52448 (15)	0.4307 (4)	0.3465 (2)	0.0524 (7)	
H2A	0.5602	0.3387	0.3398	0.063*	
C1	0.43723 (15)	0.4126 (4)	0.2986 (2)	0.0436 (6)	
H1A	0.4146	0.3068	0.2595	0.052*	

### Atomic displacement parameters (Å^2^)

**Table:**

	*U*^11^	*U*^22^	*U*^33^	*U*^12^	*U*^13^	*U*^23^
Fe1	0.01986 (15)	0.01999 (15)	0.02244 (15)	0.00008 (11)	0.00748 (11)	−0.00171 (11)
Cl1	0.0484 (3)	0.0314 (3)	0.0355 (3)	0.0008 (2)	0.0116 (3)	−0.0137 (2)
O1W	0.0454 (9)	0.0204 (7)	0.0275 (7)	0.0062 (6)	0.0188 (7)	0.0003 (6)
N6	0.0206 (8)	0.0261 (9)	0.0284 (9)	−0.0020 (7)	0.0095 (7)	0.0006 (7)
N2	0.0231 (8)	0.0186 (8)	0.0289 (9)	0.0013 (6)	0.0114 (7)	−0.0012 (7)
N1	0.0222 (8)	0.0314 (9)	0.0288 (9)	0.0024 (7)	0.0095 (7)	−0.0064 (7)
N4	0.0465 (11)	0.0195 (9)	0.0431 (11)	0.0034 (8)	0.0165 (9)	−0.0044 (8)
C11	0.0244 (10)	0.0242 (10)	0.0260 (10)	0.0006 (8)	0.0098 (8)	−0.0027 (8)
N10	0.0421 (11)	0.0280 (10)	0.0302 (9)	0.0018 (8)	0.0155 (8)	0.0042 (8)
N3	0.0358 (10)	0.0210 (9)	0.0396 (10)	0.0064 (7)	0.0186 (8)	0.0011 (8)
N7	0.0255 (8)	0.0282 (9)	0.0279 (9)	−0.0033 (7)	0.0145 (7)	0.0021 (7)
N9	0.0472 (11)	0.0292 (10)	0.0319 (10)	−0.0045 (8)	0.0229 (9)	0.0008 (8)
N8	0.0349 (10)	0.0340 (10)	0.0348 (10)	−0.0060 (8)	0.0210 (8)	−0.0001 (8)
N5	0.0390 (11)	0.0251 (10)	0.0456 (12)	−0.0034 (8)	0.0094 (9)	−0.0092 (8)
C5	0.0237 (10)	0.0301 (11)	0.0272 (10)	−0.0002 (8)	0.0080 (8)	−0.0018 (9)
C6	0.0264 (10)	0.0234 (10)	0.0266 (10)	−0.0021 (8)	0.0071 (8)	−0.0030 (8)
C12	0.0292 (10)	0.0224 (10)	0.0238 (10)	−0.0002 (8)	0.0108 (8)	−0.0005 (8)
C10	0.0280 (11)	0.0368 (12)	0.0335 (12)	0.0073 (9)	0.0071 (9)	0.0005 (10)
C4	0.0252 (11)	0.0490 (15)	0.0417 (13)	−0.0075 (10)	0.0056 (10)	−0.0078 (11)
C8	0.0263 (11)	0.0538 (16)	0.0512 (15)	−0.0093 (10)	0.0214 (11)	−0.0024 (12)
C9	0.0198 (10)	0.0519 (16)	0.0456 (14)	0.0028 (10)	0.0094 (10)	−0.0113 (12)
C7	0.0292 (11)	0.0443 (14)	0.0401 (13)	−0.0043 (10)	0.0167 (10)	0.0092 (11)
C3	0.0188 (11)	0.078 (2)	0.0485 (15)	0.0031 (12)	0.0083 (10)	−0.0018 (14)
C2	0.0288 (12)	0.0694 (19)	0.0582 (17)	0.0176 (12)	0.0168 (12)	−0.0098 (15)
C1	0.0325 (12)	0.0460 (15)	0.0503 (15)	0.0093 (11)	0.0146 (11)	−0.0157 (12)

### Geometric parameters (Å, °)

**Table:**

Fe1—O1W	1.9737 (14)	N7—C12	1.336 (3)
Fe1—N7	2.1041 (17)	N7—N8	1.337 (2)
Fe1—N2	2.1256 (16)	N9—N8	1.312 (3)
Fe1—N6	2.1602 (17)	N5—C6	1.321 (3)
Fe1—N1	2.1708 (18)	C5—C4	1.386 (3)
Fe1—Cl1	2.2308 (7)	C5—C6	1.461 (3)
O1W—H1WA	0.9774	C10—C9	1.385 (3)
O1W—H1WB	0.8241	C10—H10A	0.9300
N6—C7	1.339 (3)	C4—C3	1.377 (4)
N6—C11	1.347 (3)	C4—H4A	0.9300
N2—N3	1.331 (2)	C8—C9	1.366 (4)
N2—C6	1.334 (2)	C8—C7	1.377 (3)
N1—C1	1.340 (3)	C8—H8A	0.9300
N1—C5	1.345 (3)	C9—H9A	0.9300
N4—N3	1.313 (3)	C7—H7A	0.9300
N4—N5	1.348 (3)	C3—C2	1.368 (4)
C11—C10	1.384 (3)	C3—H3A	0.9300
C11—C12	1.461 (3)	C2—C1	1.371 (3)
N10—C12	1.322 (3)	C2—H2A	0.9300
N10—N9	1.345 (3)	C1—H1A	0.9300
			
O1W—Fe1—N7	164.20 (6)	N8—N9—N10	111.58 (17)
O1W—Fe1—N2	86.71 (6)	N9—N8—N7	107.23 (16)
N7—Fe1—N2	83.89 (7)	C6—N5—N4	103.55 (17)
O1W—Fe1—N6	91.08 (7)	N1—C5—C4	122.2 (2)
N7—Fe1—N6	76.34 (7)	N1—C5—C6	113.45 (17)
N2—Fe1—N6	90.32 (6)	C4—C5—C6	124.4 (2)
O1W—Fe1—N1	93.38 (7)	N5—C6—N2	111.68 (19)
N7—Fe1—N1	96.59 (7)	N5—C6—C5	129.50 (19)
N2—Fe1—N1	75.90 (6)	N2—C6—C5	118.81 (18)
N6—Fe1—N1	165.22 (7)	N10—C12—N7	111.92 (18)
O1W—Fe1—Cl1	94.84 (5)	N10—C12—C11	129.17 (19)
N7—Fe1—Cl1	96.38 (5)	N7—C12—C11	118.91 (18)
N2—Fe1—Cl1	171.26 (5)	C11—C10—C9	118.4 (2)
N6—Fe1—Cl1	98.24 (5)	C11—C10—H10A	120.8
N1—Fe1—Cl1	95.41 (5)	C9—C10—H10A	120.8
Fe1—O1W—H1WA	118.7	C3—C4—C5	118.4 (2)
Fe1—O1W—H1WB	121.3	C3—C4—H4A	120.8
H1WA—O1W—H1WB	119.5	C5—C4—H4A	120.8
C7—N6—C11	118.60 (17)	C9—C8—C7	119.3 (2)
C7—N6—Fe1	125.09 (15)	C9—C8—H8A	120.3
C11—N6—Fe1	116.26 (13)	C7—C8—H8A	120.3
N3—N2—C6	106.24 (16)	C8—C9—C10	119.5 (2)
N3—N2—Fe1	137.95 (13)	C8—C9—H9A	120.2
C6—N2—Fe1	115.30 (13)	C10—C9—H9A	120.2
C1—N1—C5	118.19 (19)	N6—C7—C8	122.1 (2)
C1—N1—Fe1	125.34 (15)	N6—C7—H7A	118.9
C5—N1—Fe1	116.35 (13)	C8—C7—H7A	118.9
N3—N4—N5	111.16 (17)	C2—C3—C4	119.6 (2)
N6—C11—C10	122.05 (19)	C2—C3—H3A	120.2
N6—C11—C12	113.04 (17)	C4—C3—H3A	120.2
C10—C11—C12	124.90 (19)	C3—C2—C1	119.2 (2)
C12—N10—N9	103.34 (17)	C3—C2—H2A	120.4
N4—N3—N2	107.37 (16)	C1—C2—H2A	120.4
C12—N7—N8	105.94 (17)	N1—C1—C2	122.5 (2)
C12—N7—Fe1	115.35 (13)	N1—C1—H1A	118.8
N8—N7—Fe1	138.44 (14)	C2—C1—H1A	118.8

### Hydrogen-bond geometry (Å, °)

**Table:**

*D*—H···*A*	*D*—H	H···*A*	*D*···*A*	*D*—H···*A*
O1W—H1WA···N4^i^	0.98	1.67	2.652 (2)	178
O1W—H1WB···N9^ii^	0.82	1.80	2.626 (2)	176

Symmetry codes: (i) *x*, *y*−1, *z*; (ii) *x*, −*y*+3/2, *z*+1/2.
